# Supplementary material for: Diagnostic Accuracy of Magnetic Resonance Imaging for Sagittal Cervical Spine Alignment: A Retrospective Cohort Study
Source: Int J Environ Res Public Health. 2021 Dec 10;18(24):13033. doi: 10.3390/ijerph182413033 (PMC8702200; doi:10.3390/ijerph182413033)
Supplement: Supplementary file 1 [file ijerph-18-13033-s001.zip › Table_s1.pdf]

**Table S1. Instruments used for magnetic resonance imaging.**

| Model | Manufacturer | Magnetic field strength (Tesla) |
|---|---|---|
| Skyra | SIEMENS | 3 |
| Signa HDxt | GE medical system | 1.5 |
| Signa Excite | GE medical system | 1.5 |
| Discovery MR750w | GE medical system | 3 |
| Achieva | Philips | 3 |
