# Supplementary material for: Diagnostic Accuracy of Magnetic Resonance Imaging for Sagittal Cervical Spine Alignment: A Retrospective Cohort Study
Source: Int J Environ Res Public Health. 2021 Dec 10;18(24):13033. doi: 10.3390/ijerph182413033 (PMC8702200; doi:10.3390/ijerph182413033)
Supplement: Supplementary file 1 [file ijerph-18-13033-s001.zip › Table_s2.pdf]

Table S2. Cervical alignment determined by radiography and MRI measurements

Radiography \ MRI	Cobb angle			ARA			Borden's method				
	Kyphosis	Lordosis	Straight	Kyphosis	Lordosis	Straight	Kyphosis	Lordosis	Straight	SVA>40	SVA≤40
Kyphosis	19	2	0	13	1	3	11	3	4		
Lordosis	15	48	8	5	34	41	3	24	11		
Straight	20	19	11	9	10	26	14	27	45		
SVA>40										4	1
SVA≤40										6	131

Abbreviations: MRI, magnetic resonance imaging; ARA, absolute rotational angle; SVA, sagittal vertical axis (C2-7)
