# Supplementary figures and images for: Diagnostic Accuracy of Magnetic Resonance Imaging for Sagittal Cervical Spine Alignment: A Retrospective Cohort Study
Source: Int J Environ Res Public Health. 2021 Dec 10;18(24):13033. doi: 10.3390/ijerph182413033 (PMC8702200; doi:10.3390/ijerph182413033)

**Figure S1. Head stabilizer used for magnetic resonance imaging.**

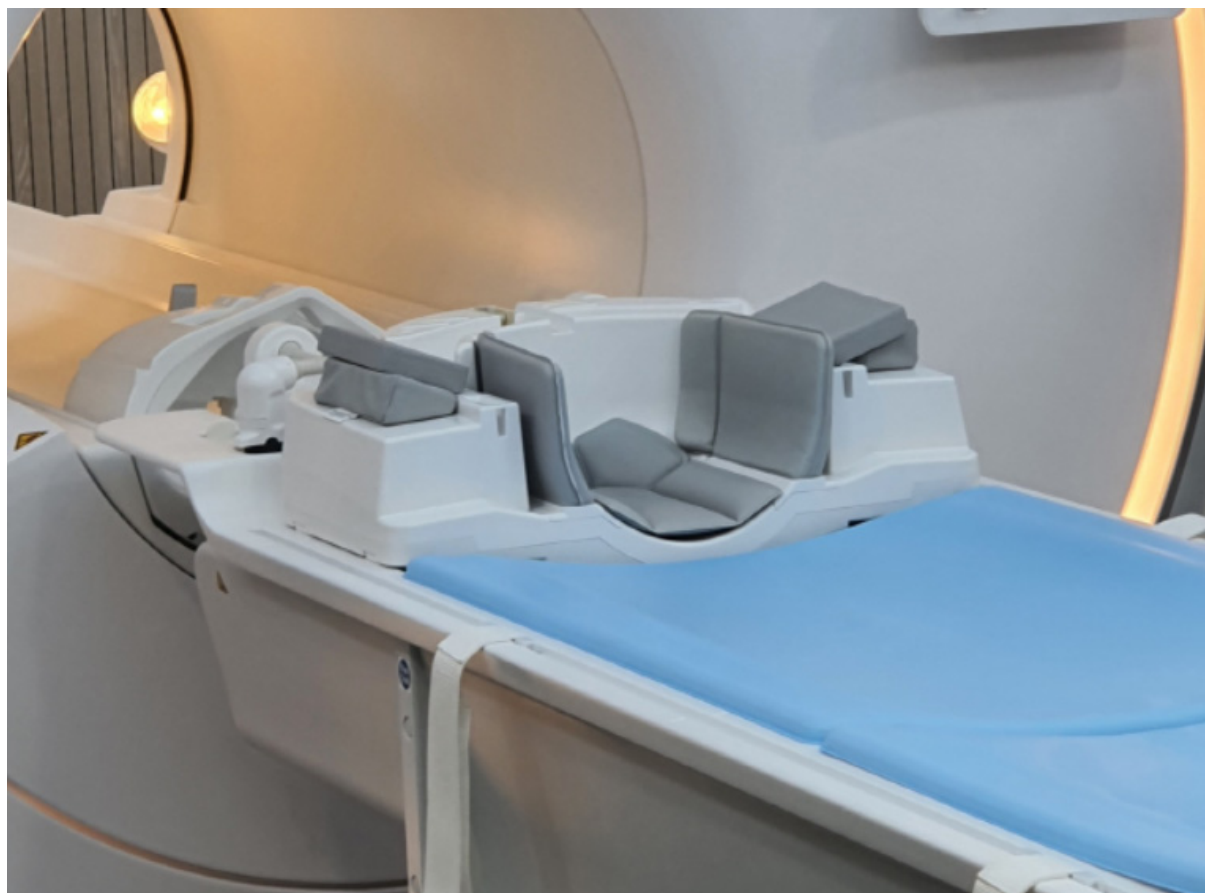

Supplement: Supplementary file 1 [file ijerph-18-13033-s001.zip › Figure_s1.pdf]

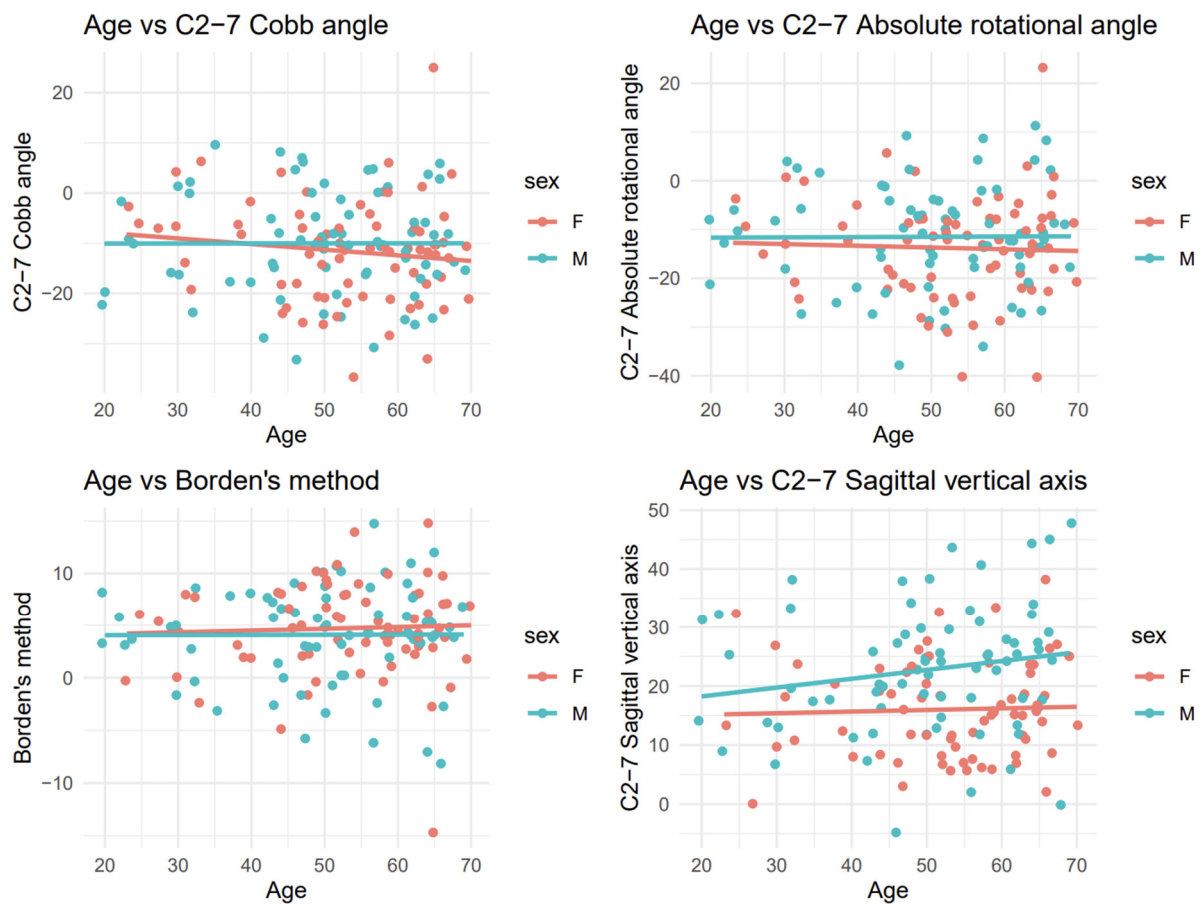

**Figure S2. Correlation between age and the four measurements**

Supplement: Supplementary file 1 [file ijerph-18-13033-s001.zip › Figure_s2.pdf]
